# Impacts of a fresh-frozen dog food on fecal consistency and microbiome in colony dogs

**DOI:** 10.1093/jas/skag303

**Published:** 2026-09-21

**Authors:** Makenzie C Melby, Claire L Timlin, Fiona B McCracken, Connie A Rojas, Catherine Carchedi, Brittany White, Lindsay Cassibry, Amanda Blake, Craig N Coon

**Affiliations:** Four Rivers Kennel, Walker, MO 64772, United States; Four Rivers Kennel, Walker, MO 64772, United States; Four Rivers Kennel, Walker, MO 64772, United States; Animal Biome, Oakland, CA 94609, United States; Ollie Pets Inc, Reno, NV 89511, United States; Ollie Pets Inc, Reno, NV 89511, United States; Ollie Pets Inc, Reno, NV 89511, United States; Gastrointestinal Laboratory, Texas A&M University, College Station, TX 77843, United States; Four Rivers Kennel, Walker, MO 64772, United States

**Keywords:** dog nutrition, short-chain fatty acids, 16S rRNA gene sequencing

## Abstract

Fresh-frozen dog diets are becoming increasingly popular among pet owners, but little is known regarding the impact of this diet on canine gut health and the microbiome. This study evaluated the impacts of a fresh-frozen versus an extruded kibble diet on the fecal consistency, metabolites, and microbiome in 16 adult Labrador Retrievers (average age 4.2 ± 0.26 yr; average weight: 25.7 ± 3.18 kg) over the course of 6 months. Dogs were randomly assigned to one of the two diets in a parallel design. Feces were collected at baseline, 3 months, and 6 months for short-chain fatty acid, ammonia, and microbiome analyses. An additional sample was collected at 1 mo for microbiome analysis. Fecal microbiome was analyzed via 16S rRNA gene sequencing. Fecal consistency scores were recorded daily, and body weights and body condition scores were recorded weekly. Data were analyzed across time and treatments using a repeated measures analysis of variance. Results showed that fecal consistency was improved in the dogs fed the fresh diet, with differential changes detected shortly after the transition phase onto the test diets (*P* < 0.01). Fecal butyrate concentrations were greater in the fresh group (*P* < 0.01), and fecal valerate concentrations fluctuated more in the kibble group while the fresh group remained more consistent over time (*P* = 0.05). No differences were observed in fecal moisture, pH, ammonia, or other fecal short-chain or branched-chain fatty acids. General dog health was not negatively impacted by either diet, as observed through weight maintenance and veterinary wellness exams. Diet type impacted fecal microbiome composition, as shown by differences in beta diversity between the fresh and kibble groups (*P* < 0.01). In addition, microbiomes of dogs fed a fresh diet were enriched in short-chain fatty acid producers such as *Turicibacter sanguinis, Ruminococcus*, and *Clostridium* compared to dogs fed kibble. Overall, the fresh diet increased fecal consistency and fecal butyrate, although other fecal parameters were not different.

## Teaser

A fresh-frozen dog diet improved fecal quality compared to an extruded kibble diet. Diet type also influenced fecal microbiome composition.

## Introduction

Pet owners see their pets as family members and want to keep their pets in good health. Interest in pet foods that consumers view as healthier is growing, and many dog owners are seeking alternatives to extruded kibble. As a result, fresh dog food, also referred to as fresh-frozen or gently cooked, is one of the fastest-growing sectors of the pet food industry (Pet Food in the US Market Research Report | Packaged Facts). Due to fresh dog food’s increasing popularity, more research is being conducted investigating its effects on canine health. While some owners are moving toward homemade dog food, commercially produced fresh-frozen foods may provide advantages over these diets by ensuring nutritional completeness of the diets, as homemade diets can often lack vital nutrients (O’Brien et al. 2025). Growing interest in fresh-frozen dog diets also coincides with growing interest in the gut microbiome, which can be modulated by diet (Sun and Zhai 2025).

The gut microbiome plays a critical role in dogs’ health and impacts many body systems. Gut microbiota support the immune system by preventing pathogens from colonizing the gut. Some microbes produce compounds that are toxic to pathogenic bacteria; for example, beneficial *Escherichia coli* in the gut produce bacteriocin, which inhibits the growth of pathogenic strains of *E. coli* (Sassone-Corsi et al. 2016). Gut microbes also influence their environment by producing pH altering compounds, such as short-chain fatty acids (SCFA), which make the gut inhospitable to pathogens (Kamada et al. 2013). In addition to helping inhibit pathogens, SCFA are an energy source for intestinal epithelial cells and promote gut motility (Suchodolski 2022).

Several studies have observed a greater apparent total tract digestibility of fresh dog food compared to kibble, meaning dogs can absorb more nutrients and energy from fresh food (Algya et al. 2018; Do et al. 2021; Tanprasertsuk et al. 2021; Geary et al. 2024). Few studies have investigated the impact of a fresh diet on the gut microbiome, and those that have reported conflicting results. While a fresh diet impacted certain bacterial taxa in a consistent way across studies, other taxa were elevated in some findings, depressed in others, or similar to that of dogs fed kibble in others (Algya et al. 2018; Geary et al. 2024; Oba et al. 2025; Yang et al. 2025). Most previous studies investigating the effects of a fresh diet on the gut microbiome of dogs have used Beagles. Previous work indicating differences in the gut microbiome of different breeds and dogs of different sizes highlights the need for microbiome studies in other dog breeds (Reddy et al. 2019; You and Kim 2021; Deschamps et al. 2022).

The objective of this article was to evaluate the impacts of fresh-frozen food versus extruded kibble on the fecal health and microbiome of dogs. Changes in fecal quality and fecal microbiome composition of Labrador Retrievers were evaluated across a 6-mo feeding period. The authors hypothesized that fresh-frozen food would not be detrimental to the dogs’ health and that dogs fed fresh-frozen food would have an altered fecal microbiome compared to dogs fed kibble.

## Materials and methods

All experimental procedures were approved by the Institute of Animal Care and Use Committee at Four Rivers Kennel under Protocol FRK-67. Dogs were randomly assigned to either a fresh-frozen diet or a dry extruded kibble diet and fed for 184 d, including transition onto the test diet. Fecal samples were collected at baseline, 3 months, and 6 months, with an additional sample collected at 1 mo solely for fecal microbiome analysis. The feeding period began in December 2024 and ended in June 2025.

### Animals and housing

Sixteen intact adult Labrador Retrievers (eight male and eight female), averaging 4.2 ± 0.26 yr old and 25.7 ± 3.18 kg at time of enrollment, were enrolled in this 6-mo study. All dogs were individually housed in temperature-controlled kennels made of galvanized chain-link fencing (1.2 m × 1.2 m × 1.8 m) with concrete flooring and had access to outdoor socialization yards for 6-8 h per day dependent on weather. All animals had free access to automatic waterers in both the kennels and the yards. All animals were up to date on vaccinations and received monthly prophylactic heartworm and parasite prevention. Normal schedules for preventatives were maintained during the course of the study, and yearly vaccines were given after completion of the mid-study sampling. Body weights and body condition scores (BCS) were measured weekly on each animal. Veterinary wellness exams were performed by a licensed veterinarian on d 0 and 182 which consisted of: visual examination of the teeth, eyes, ears, and coat; palpation of the abdomen, lymph nodes, and limbs; and examination of the heart and lungs with a stethoscope.

### Diets

Dogs were blocked by age, sex, BCS, and weight and randomly assigned to either: 1) fresh-frozen diet (fresh; Ollie Chicken Dish with Carrots recipe; *n* = 8), or 2) commercial dry extruded kibble (kibble; Blue Buffalo Life Protection Chicken and Brown Rice Recipe; *n* = 8) in a parallel design. The fresh-frozen diet was cooked to achieve pathogen lethality (>161°F), rapidly cooled, packaged, and frozen for long-term storage. Before feeding, all diets were tested to confirm compliance with nutritional and microbiological standards, including Salmonella spp., *Listeria monocytogenes*, aerobic plate count, total coliforms, and generic *E. coli*. Fresh-frozen food was thawed in a refrigerator for up to 3 d before feeding. Before the study and during the baseline sampling, dogs were maintained on the basal kennel diet (MFA Gold-N Pro, MFA Incorporated, Columbia, MO; dietary information in Supplementary Material S1). Dogs were fed at amounts based off previous kennel records for weight maintenance and transitioned to the new food with the following regimen based on total daily calories: d 0, 1—75% basal kennel diet, 25% assigned diet; d 2, 3—50% basal kennel diet, 50% assigned diet; d 4, 5—25% basal kennel diet, 75% assigned diet. All dogs had ad libitum access to water. For dogs fed the kibble diet, males were offered on average 692 ± 85 g (89.1 ± 9.75 kcal/kg BW) of treatment diet per day, and females were offered 608 ± 129 (88.8 ± 12.2 kcal/kg BW) g per day. For dogs fed the fresh diet, males were offered on average 1,862 ± 152 g (87.7 ± 10.4 kcal/kg BW) of treatment diet per day, and females were offered 1,483 ± 175 g (79.8 ± 17.9 kcal/kg BW) per day. The diets were weighed out in grams each day and dogs were provided at least 45 min to consume their portions. Any food not consumed was collected and weight of orts was recorded. Proximate analysis and ingredients of diets are listed in Table 1. Dogs were fed tests diets for 184 d, including the transition period.

**Table 1:** Analyzed chemical composition of test diets.

Item	Kibble^1^	Fresh^2^
**Dry matter, DM, %**	91.81	28.80
**DM basis**		
** Protein (crude), %**	28.10	37.50
** Fat, %**	15.58	19.44
** Fiber (crude), %**	4.10	2.95
** Total dietary fiber, %**	14.49	9.03
** Insoluble fiber, %**	9.26	5.56
** Soluble fiber, %**	5.23	3.47
** Ash, %**	6.95	4.47
**Grams/1,000 kcal**		
** Protein (crude)**	71.01	83.08
** Fat**	39.37	43.07
** Fiber (crude)**	10.36	6.54
** Total dietary fiber**	36.62	20.01
** Insoluble fiber**	23.40	12.32
** Soluble fiber**	13.22	7.69
** Ash**	17.56	9.90

1 Kibble diet (Blue Buffalo Life Protection Chicken and Brown Rice Recipe): deboned chicken, chicken meal, brown rice, oatmeal, barley, peas, chicken fat (preserved with mixed tocopherols), dried tomato pomace, natural flavor, flaxseed (source of omega 3 & 6 fatty acids), dried yeast, salt, direct dehydrated alfalfa pellets, potassium chloride, dried chicory root, alfalfa nutrient concentrate, calcium carbonate, choline chloride, L-Threonine, DL-Methionine, preserved with mixed tocopherols, dried sweet potatoes, carrots, taurine, zinc amino acid chelate, zinc sulfate, vegetable juice for color, ferrous sulfate, vitamin e supplement, iron amino acid chelate, glucosamine hydrochloride, blueberries, cranberries, barley grass, parsley, turmeric, dried kelp, Yucca Schidigera extract, niacin (Vitamin B3), calcium pantothenate (Vitamin B5), copper sulfate, biotin (Vitamin B7), L-ascorbyl-2-polyphosphate (source of Vitamin C), L-Lysine, Vitamin A supplement, copper amino acid chelate, manganese sulfate, manganese amino acid chelate, thiamine mononitrate (Vitamin B1), riboflavin (Vitamin B2), Vitamin D3 supplement, Vitamin B12 supplement, pyridoxine hydrochloride (Vitamin B6), calcium iodate, folic acid (Vitamin B9), sodium selenite, oil of rosemary.

2 Fresh diet (Ollie Chicken Dish with Carrots): chicken, rice, carrots, chicken livers, peas, potatoes, spinach, cranberries, tricalcium phosphate, salmon oil, salt, taurine, zinc gluconate, Vitamin E supplement, ferrous sulphate, copper amino acid chelate, manganese amino acid chelate, cholecalciferol (Vitamin D3) supplement, riboflavin (Vitamin B2), thiamine hydrochloride (Vitamin B1), pyridoxine hydrochloride (Vitamin B6), potassium iodide.

### Fecal consistency scoring

Fecal consistency was recorded daily for the duration of the trial. Kennel technicians were trained and evaluated for proper fecal scoring before beginning the trial to ensure consistency. Feces were scored based on what each dog had produced overnight in individual kennels and during fresh collections. Scores from multiple defecations were averaged. If a dog did not produce a fecal sample overnight, the dogs were accompanied outside individually to obtain a score. Feces were scored as follows: 1) liquid diarrhea, no form; 2) loose feces, mix of formed and unformed; 3) partially formed feces, very moist and soft; 4) firm, well-formed, easy to pick up; and 5) little to no moisture, hard, crumbled easily.

### Fecal sample collection and analysis

Fresh fecal samples were collected from each dog at baseline (d −6), 3 months (d 88–91), and 6 months (d 183–185). An additional sample was collected at the 1-mo mark (d 29 and 30) for microbiome analysis. A single fresh sample was collected within 30 min of defecation, but obtaining collections on all dogs took up to 3 d to complete. Samples were immediately processed into aliquots. A pea sized sample from the interior of the feces was collected and transferred into a 2 mL screw-cap tube containing 1.4 mL of nucleic acid preservation buffer to stabilize and preserve microbial DNA (Animal Biome, Oakland, CA) and stored at room temperature. Another aliquot was stored at −80 °C in polypropylene cryovials for analysis of SCFA at the Gastrointestinal Laboratory at Texas A&M University (College Station, TX). A fecal extract was prepared by homogenizing 2 g of feces with 4 mL of distilled water with a vortex for 30 s; pH was then measured using a calibrated pH meter. Tubes were then centrifuged at 2,500 × *g* for 20 min at room temperature and supernatant was collected and stored at −80 °C until ammonia analysis using a rapid ammonia assay kit (K-AMIAR, Neogen, Lansing, MI). Samples for ammonia were further diluted with distilled water for a total dilution of 1:40. Lastly, the remainder of collected samples were frozen at −80 °C. Samples were later thawed overnight at 4 °C and moisture by loss on drying was analyzed by drying between 2 and 5 g in an oven at 133 °C for 4 h.

### Short-chain fatty acid analysis

Fecal concentrations of SCFA (acetate, propionate, butyrate, valerate) and branched chain fatty acids (isobutyrate, isovalerate) were measured using a gas chromatography-mass spectrometry assay as previously described with modifications (Minamoto et al. 2019). Feces were weighed, and 2N hydrochloric acid was added using 4 parts liquid to 1 part feces. Samples were then homogenized in a multitube vortexer for 30 min at high speed at room temperature followed by centrifugation at 3,000 × *g* for 20 min at 5 °C. Supernatants were collected using serum filters (FILTER SAMPLER Blood Serum Filters, Porex Corporation, Fairburn, Georgia, USA). Sample acid extracts were stored at −80 °C until analysis.

Mixed standards were prepared with serial dilutions in 2 N hydrochloric acid to 11 levels ranging from 0.4 to 40 mM (acetate and propionate), 0.01–10 mM (isobutyrate, butyrate, and isovalerate), and 0.002–2.5 mM (valerate).

For solid-phase extraction sample clean-up, 250 µL of each sample acid extract or standard level was combined with 25 µL internal standard mix (200 mM d4-acetic acid, 50 mM d5-propionic acid, 25 mM d7-butyric acid, 781.25 µM d9-valeric acid in water) and loaded onto C18 columns (Discovery DSC-18 SPE Tube, Supelco, Bellefonte, Pennsylvania, USA) on vacuum manifold. Next, analytes were eluted with the addition of 1 mL diethyl ether, and an additional 1 mL diethyl ether was added to each sample. In a glass test tube, 250 µL of the diluted eluate was combined with 50 µL derivatization reagent (*N*-tert-butyldimethylsilyl-*N*-methyltrifluoroacetamide, MTBSTFA) and 250 µL diethyl ether and incubated at room temperature for 60 min. Samples were vortexed and 80 µL transferred to glass autosampler vial inserts for analysis.

A gas chromatograph (Agilent 7890A, Agilent Technologies Inc., Santa Clara, California, USA) equipped with a DB-1 MS capillary column (Agilent Technologies Inc.) coupled with an electron ionization mass spectrometer (Agilent 5975C, Agilent Technologies Inc.) was used for chromatographic separation and quantification. Injection volume was set to 2 µL with a 5:1 split ratio, 250 °C inlet temperature, and helium carrier gas. Gas chromatograph temperature program was as previously described (Minamoto et al. 2019). The mass spectrometer was operated in positive-ion mode with selective ion monitoring of mass-to-charge ratios (m/z) of the following: 117 (acetate), 120 (d4-acetate), 131 (propionate), 136 (d5-propionate), 145 (isobutyrate and butyrate), 152 (d7-butyrate), 159 (valerate and isovalerate), and 168 (d9-valerate). Those analytes with the same m/z were baseline resolved chromatographically.

Analysis was performed in Agilent MassHunter Quant (v10.0) software. Standard curves were generated from prepared mixed standards and applied to unknowns to calculate concentrations. Final concentrations were adjusted for fecal dry matter (DM) and expressed as mol/g fecal dry matter. Fecal DM values for SCFA analysis were obtained by lyophilization of 0.2–1 g feces.

The assay modifications were partially validated in canine feces for accuracy and precision following ICH M10 guidelines for bioanalytical method validation (ICH M10 on bioanalytical method validation—Scientific guideline | European Medicines Agency (EMA) 2019). Standards fell within 20% of expected values, and five canine fecal samples analyzed consecutively four times had coefficients of variation less than 8% when analyzed on the same day and less than 12% when analyzed on 4 different days within a 6-d period. While a full validation covering all parameters (such as comprehensive stability testing and matrix effect profiling) was not performed in this study, stability and spiking recovery were performed in the same lab for a prior study of canine feces for SCFA acetate, propionate and butyrate (Minamoto et al. 2019). The cross-validation testing conducted was sufficient to demonstrate the modified method is robust and fit for its intended purpose within the scope of this study.

### Food and fecal parameters statistical analysis

Statistical analysis of food consumption and fecal parameters was performed in SAS Studio 3.8 (SAS Institute, Cary, NC). Food consumption data was analyzed as a proportion of food consumed to food offered and in total grams consumed. For both food consumption and fecal consistency data, measurements and scores were averaged by week for statistical analysis. Days of transition onto diet were excluded in analysis of weekly food consumption data. Fecal consistency data was also analyzed on a daily basis for the first 10 d of study including transition onto the diets. Other fecal parameters (moisture, pH, ammonia, SCFA, branched chain fatty acids (BCFA)) were analyzed at three timepoints (baseline and 3 and 6 months). Box Cox data transformations were applied when necessary to maintain the normality of residuals assumption.

All variables were analyzed using a repeated measures mixed model (PROC MIXED) with Tukey–Kramer post-hoc adjustment in SAS Studio v3.82 (SAS Institute Inc., Cary, NC). Body weight and BCS models included fixed effects of sex, time, diet, diet*time, and diet*sex interactions. Non-significant diet*sex effects were removed from models. All other data were analyzed using repeated measures mixed models with fixed effects of diet, time, diet*time interaction with sex as a random variable. Dog within diet was specified as the repeated subject for all models. Covariance structure selection for these models was based on variance and covariances of the unstructured model and the lowest corrected Akaike information criterion (AICc). Significance was set at *P* ≤ 0.05 and tendencies were set at 0.05 < *P* ≤ 0.08. Data is presented as least square means with their standard errors (SEM). Transformed data are presented as the back-transformed means and SEM estimations according to Jorgensen and Pedersen (1998).

### Microbiome sequencing and bioinformatics

DNA was extracted from 0.5 g of fecal material using the QIAGEN DNeasy PowerSoil Pro Kit (QIAGEN, CA, USA) per the manufacturer’s protocol.

The length of the 16S rRNA gene (V1-V9) was amplified via PCR using universal primers 27F (5′-AGRGTTYGATYMTGGCTCAG-3′) and 1492R (5′-RGYTACCTTGTTACGACTT-3′), each appended with unique 16-bp asymmetric barcodes to enable multiplexing. Amplified products were purified and sequenced on a PacBio Sequel IIe instrument (Pacific Biosciences, CA, USA). The Zymo Microbial Community DNA Standard was included as a positive control, and nuclease-free water served as a negative control.

Raw PacBio sequence data were processed into circular consensus (HiFi) reads using SMRT Analysis software (v11.0.0). Quality control and amplicon sequence variant (ASV) inference were performed in QIIME 2 using the DADA2 plugin. Reads were trimmed of adapters and filtered to retain sequences between 1,300 and 1,600 bp. De-noising was conducted using the pseudo-pooling approach, and chimeric sequences were identified and removed using pooled chimera detection.

Taxonomic classification of ASV sequences was done within QIIME2, using a combination of the Naive Bayes (sklearn) classifier and VSEARCH, with the SILVA reference database (v138.1 NR99). Confidence thresholds were set to 0.7 for the sklearn classifier and 0.99 for VSEARCH to improve assignment specificity. The resulting ASV table was collapsed to the species level and imported into R (v4.3.0) for downstream statistical analyses.

Highly reproducible and low technical variability microbiome sequencing occurred in three batches (Rojas et al. 2026).

### Microbiome statistical analysis

Plots showcasing the relative abundances (%) of top bacterial genera and top bacterial species were constructed in R using the ggplot2 package. Wilcoxon rank-sum tests evaluated whether the relative abundance of bacterial species differed between dogs fed a kibble diet versus a fresh diet at 1, 3, and 6 months post-diet transition. *P*-values were adjusted for multiple comparisons using the False Discovery Rate (FDR). Species tested included all taxa with a mean relative abundance >0.8% across samples, in addition to five taxa previously identified as core members of the healthy canine gut microbiome (Rojas et al. 2024): [*Ruminococcus*] *gnavus, Faecalimonas umbilicata, Lachnoclostridium* UC, *Lachnospiraceae* UC, and *Sutterella stercoricanis.*

For alpha diversity analyses, samples were rarefied to the minimum sequencing depth (2,266 reads) to account for variation in library size. Alpha diversity was then quantified using Chao1 richness, Shannon diversity, and Pielou’s evenness using the phyloseq and microbiome (v1.30.0) packages. Generalized linear models compared microbiome diversity between diet groups (kibble vs. fresh) at baseline and at 1, 3, and 6 months post-diet transition. The effects of transitioning diets on microbial diversity was analyzed using a linear mixed-effects model including timepoint, diet, and their interaction as fixed effects, with dog ID included as a random effect to account for repeated measures. This approach allowed us to test whether diversity shifted when the diet changed from MFA Gold-N Pro to the kibble or fresh diet.

Differences in the microbiome composition between dogs fed a kibble diet versus a fresh diet at baseline and at 1, 3, and 6 months post-diet transition were evaluated using Permutational Multivariate Analysis of Variance (PERMANOVA). Each model was run with the vegan package (v.2.7-1) with 999 permutations and used both Bray-Curtis dissimilarities (based on relative abundances) and Aitchison distances (based on centered log-ratio [CLR]–transformed abundances).

Differential abundance testing using the LinDA package (v0.2.0) was conducted to further examine diet-associated divergence in the canine gut microbiome. Analyses were performed on CLR-transformed species-level abundances, restricting the dataset to taxa present in at least 33% of samples. Separate models were fit for the 3-mo and 6-mo timepoints. Species identified as significantly different between diet groups were visualized as log₂ fold changes using ggplot2.

## Results

### Body weight and body condition

Body weight and body condition score results are presented in Table S1 and Figures S1 and S2 (see online supplementary material for a color version of these figures). Body weights and BCS remained in the healthy range for all dogs during the study. There were no differences due to diet or diet*time interaction on body weights (*P* = 0.82). Body weights and BCS did fluctuate slightly across weeks (*P* < 0.01), particularly in the second half of the study as the seasons shifted from winter to early summer and temperatures increased, but weights from wk 0 to 26 did not differ by post-hoc analysis (*P* = 0.82). There were no effects of diet on BCS (*P* ≥ 0.09).

### Fecal consistency scores

Substantial differences between treatments were noted in the fecal consistency scores between the two treatments in the first week (wk 0, d 0–6) of the study during the diet transition. To ensure that the groups were not significantly different from each other at baseline, analysis of fecal scores was performed using only the first 11 d of data (d 0–10 of the study). The groups’ scores were similar on d 0 before diet transition (fresh: 2.89 ± 0.26; kibble: 3.04 ± 0. 24; post-hoc *P *= 1.00), before gradually diverging within the first 10 d (*P *< 0.01, Figure 1A), influencing the overall treatment (*P *< 0.01) and time effects (*P *= 0.08). When analyzed as a weekly average, fecal consistency scores were greater in the fresh diet group (3.73 ± 0.10) compared to kibble fed dogs (2.71 ± 0.13; *P* < 0.01), indicating improved consistency. There were also significant effects of time and diet*time interaction (*P* < 0.01), but no apparent trends in the diets over time were observed (Figure 1B).

**Figure 1:**
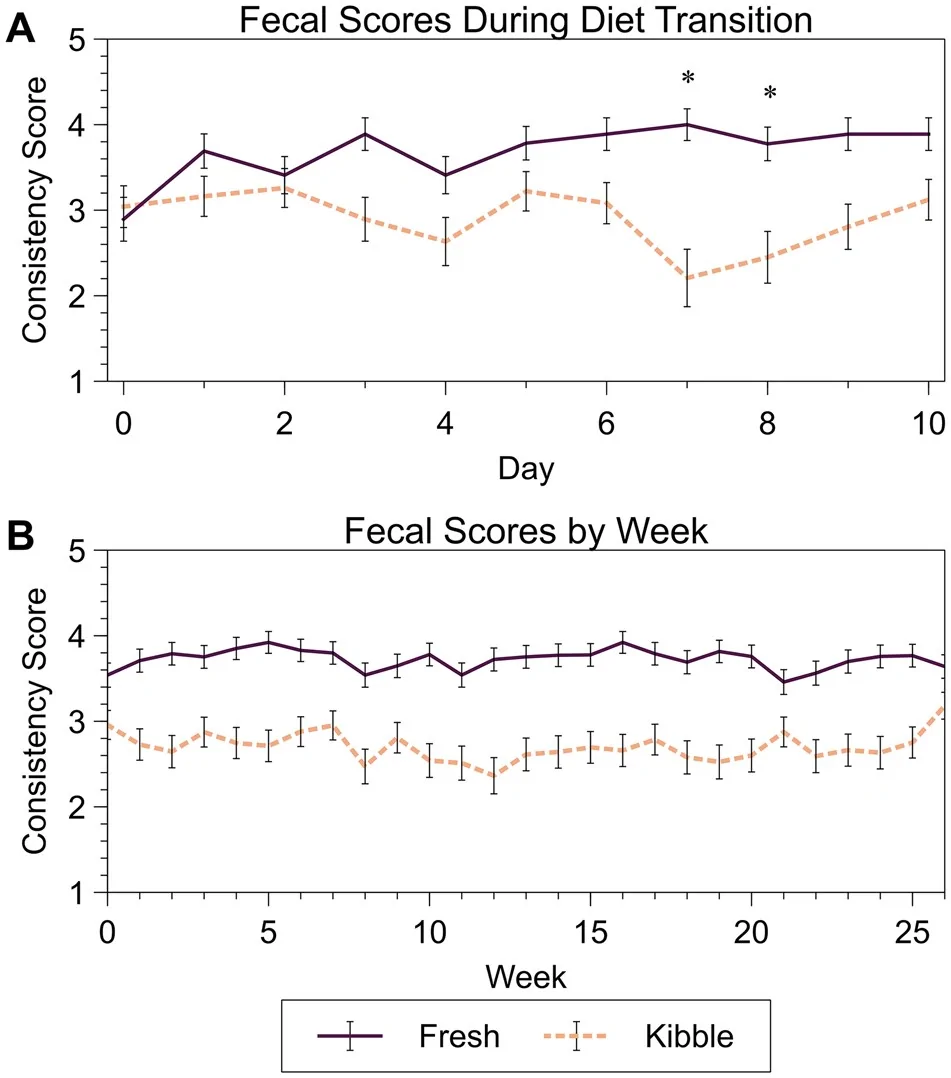
Fecal consistency scores A) during the first 10 d of study including diet transition and B) as weekly averages over the course of the 26-wk. Week 0 represents the week of diet transition (study d 0–6). *Denote significant differences between diets (*P* < 0.05) for a given day.

### Fecal parameters

Results for fecal parameters and SCFA are presented in Table 2. There were no differences in fecal moisture between diets or diet*time interaction (*P* ≥ 0.35). Moisture did increase overall by end of study in both groups (*P* = 0.01). Fecal pH was not affected by diet, time, or diet*time interaction (*P* ≥ 0.13). Fecal ammonia was not affected by diet (*P* ≥ 0.17) but did decrease after 3 months and was intermediate to baseline by 6 months (*P* = 0.04).

**Table 2:** Results for fecal analytes by diet and timepoint.

Item	Month	Diet		*P-*value		
		Kibble	Fresh	Diet	Time	Diet × Time
**Moisture, %**	0^b^	71.8 ± 1.01	71.4 ± 1.01	0.59	0.01	0.35
	3^ab^	73.9 ± 2.06	73.3 ± 2.06			
	6^a^	73.5 ± 2.03	76.9 ± 2.03			
**pH**	0	6.51 ± 0.157	6.37 ± 0.157	0.13	0.96	0.27
	3	6.28 ± 0.157	6.55 ± 0.157			
	6	6.27 ± 0.157	6.65 ± 0.157			
**Ammonia, g/L**	0^a^	0.21 ± 0.03	0.27 ± 0.05	0.17	0.04	0.60
	3^b^	0.14 ± 0.02	0.16 ± 0.03			
	6^ab^	0.19 ± 0.03	0.19 ± 0.03			
**Acetic acid, µmol/g DM**	0	368.12 ± 31.57	400.41 ± 34.34	0.14	0.18	0.15
	3	328.12 ± 40.05	330.96 ± 40.34			
	6	336.50 ± 25.92	423.56 ± 32.63			
**Propionic acid, µmol/g DM**	0^b^	165.79 ± 18.95	188.33 ± 18.95	0.49	<0.01	0.63
	3^ab^	203.33 ± 18.95	191.01 ± 18.95			
	6^a^	212.53 ± 18.95	237.77 ± 18.95			
**Isobutyrate, µmol/g DM**	0	6.13 ± 0.91	7.16 ± 0.91	0.43	0.90	0.63
	3	6.80 ± 0.91	6.78 ± 0.91			
	6	6.42 ± 0.91	7.64 ± 0.91			
**Butyrate, µmol/g DM**	0	49.51 ± 8.08	63.36 ± 10.33	<0.01	0.14	0.20
	3	47.44 ± 7.74	66.36 ± 10.82			
	6	49.30 ± 8.04	92.30 ± 15.05			
**Isovalerate, µmol/g DM**	0	6.51 ± 0.89	7.40 ± 0.89	0.37	0.81	0.81
	3	6.31 ± 0.89	6.62 ± 0.89			
	6	6.12 ± 0.89	7.39 ± 0.89			
**Valerate, µmol/g DM**	0	0.88 ± 0.29^b^	1.54 ± 0.67^ab^	0.88	0.10	0.05
	3	3.22 ± 2.05^a^	1.41 ± 0.59^ab^			
	6	1.45 ± 0.62^ab^	1.32 ± 0.54^ab^			

a,b Differing superscripts within the month column denote significant differences between time points (*P* < 0.05). Differing superscripts within and across the diet columns denote significant (*P* < 0.05) differences within diet × time interaction.

**Table 3:** Bacterial abundances at four timepoints.^1^

Phylum	Family	Species	Timepoint	Kibble (mean)	Fresh (mean)	*P*-value	Adjusted *P*-value
**Actinobacteriota**	Coriobacteriaceae	*Collinsella intestinalis*	Baseline	1.5308	0.9014	0.2936	0.8542
			Month 1	1.0564	0.6386	0.9163	0.9774
			Month 3	2.0726	1.9255	0.7527	0.9199
			Month 6	3.0484	1.506	0.5995	0.8286
**Bacteroidota**	Bacteroidaceae	*Bacteroides UC*	Baseline	0.9283	2.3532	1	1
			Month 1	4.4202	11.3118	0.1152	0.4095
			Month 3	3.7398	10.3354	0.7527	0.9199
			Month 6	2.8778	6.3384	0.0742	0.3958
	Bacteroidales UC	*Bacteroidales UC*	Baseline	0.3337	0.2699	0.6662	0.8633
			Month 1	1.4275	0.1706	0.0523	0.2787
			Month 3	0.9765	2.1698	0.957	0.9879
			Month 6	3.1559	0	0.0273	0.2194
	Prevotellaceae	*Alloprevotella rava*	Baseline	0.4006	0.2933	0.5984	0.8633
			Month 1	3.0202	2.085	0.0929	0.4095
			Month 3	2.4963	2.8547	0.2076	0.4428
			Month 6	2.9756	1.1216	0.1722	0.5509
		*Prevotella copri*	Baseline	1.3042	1.2723	0.4945	0.8633
			Month 1	15.3835	10.7585	0.4008	0.7847
			Month 3	12.3456	1.7805	0.0023	0.0248
			Month 6	8.895	0.3813	0.169	0.5509
**Firmicutes**	Clostridiaceae	*Clostridium disporicum*	Baseline	3.2725	1.9279	0.3446	0.8633
			Month 1	0.0091	0.8895	0.0347	0.2776
			Month 3	0.0089	0.3684	0.2275	0.4536
			Month 6	0.086	0.0708	0.5377	0.8286
		*Clostridium perfringens*	Baseline	3.5066	0.1656	0.203	0.7218
			Month 1	0.0867	7.6431	0.0109	0.1162
			Month 3	0.0305	0.7129	0.0283	0.1264
			Month 6	0.0879	10.7897	0.0006	0.0133
	Erysipelatoclostridiaceae	*Catenibacterium mitsuokai*	Baseline	1.4996	0.2393	0.1403	0.7218
			Month 1	5.0883	0.137	0.0031	0.0727
			Month 3	5.5361	0.0931	0.0008	0.0248
			Month 6	6.2393	0.0131	0.0008	0.0133
	Erysipelotrichaceae	*Turicibacter sanguinis*	Baseline	29.5828	25.3942	0.5995	0.8633
			Month 1	1.4795	1.698	0.8336	0.9774
			Month 3	2.1907	5.9398	0.0356	0.1264
			Month 6	1.0898	1.0054	0.7509	0.8286
	Lachnospiraceae	*[Ruminococcus] gnavus*	Baseline	0.4011	1.092	0.4428	0.8633
			Month 1	0.4847	0.462	0.9157	0.9774
			Month 3	1.0237	1.4014	0.8336	0.9199
			Month 6	0.8584	0.5675	0.3446	0.6891
		*Blautia hansenii*	Baseline	0.7911	0.7324	1	1
			Month 1	0.6678	0.8899	0.2466	0.5988
			Month 3	0.6924	1.5047	0.4306	0.6724
			Month 6	0.7399	1.8097	0.2922	0.6264
		*Faecalimonas umbilicata*	Baseline	0.1704	0.4253	0.5898	0.8633
			Month 1	0.1474	0.2206	1	1
			Month 3	0.1941	0.4373	0.241	0.4536
			Month 6	0.228	0.3175	0.6275	0.8286
		*Lachnoclostridium UC*	Baseline	0.0588	0.0438	0.3983	0.8633
			Month 1	0.0506	0.1727	0.197	0.5535
			Month 3	0.0598	0.2771	0.8292	0.9199
			Month 6	0.1145	0.2528	0.7422	0.8286
		*Lachnospiraceae UC*	Baseline	0.2997	0.317	0.7059	0.8688
			Month 1	0.144	0.0934	0.4504	0.7847
			Month 3	0.1642	0.1517	0.7509	0.9199
			Month 6	0.2347	0.8681	1	1
	Lactobacillaceae	*Lactobacillus acidophilus*	Baseline	0.2165	0.9356	0.0565	0.7218
			Month 1	7.1533	10.09	0.8336	0.9774
			Month 3	8.7663	0.3283	0.0587	0.1708
			Month 6	4.8528	0.7179	0.0929	0.4041
		*Lactobacillus animalis*	Baseline	1.6754	5.8382	0.5984	0.8633
			Month 1	7.2046	2.7829	0.2076	0.5535
			Month 3	4.1217	1.9283	0.2936	0.522
			Month 6	1.7075	1.8582	0.2072	0.6029
		*Lactobacillus johnsonii*	Baseline	0.0068	0.1371	0.3714	0.8633
			Month 1	5.5789	4.4046	0.5992	0.872
			Month 3	0.7522	0.0367	0.0881	0.2349
			Month 6	0.3787	0.0973	0.2809	0.6264
		*Lactobacillus phage*	Baseline	1.339	0.2395	0.6137	0.8633
			Month 1	1.0691	0.1113	0.262	0.5988
			Month 3	2.5534	0.3212	0.0343	0.1264
			Month 6	1.9312	3.764	0.7509	0.8286
		*Lactobacillus reuteri*	Baseline	0.6655	0.8455	0.0946	0.7218
			Month 1	10.6683	8.3515	0.4622	0.7847
			Month 3	8.849	1.7087	0.1415	0.3234
			Month 6	13.3922	3.0626	0.5995	0.8286
		*Lactobacillus salivarius*	Baseline	2.1806	0.2977	0.5107	0.8633
			Month 1	1.0488	0.1381	0.9127	0.9774
			Month 3	3.2447	0.7733	0.1412	0.3234
			Month 6	1.4935	4.4178	0.4622	0.7847
	Peptococcaceae	*Peptococcus UC*	Baseline	0.6144	0.39	0.6744	0.8633
			Month 1	1.1925	0.2055	0.0045	0.0727
			Month 3	0.9579	1.5203	0.8335	0.9199
			Month 6	1.3548	1.0189	0.101	0.4041
	Peptostreptococcaceae	*[Clostridium] dakarense*	Baseline	5.5507	4.8782	0.9164	1
			Month 1	0.0106	0.1835	0.1325	0.4239
			Month 3	0.0525	0.8833	0.002	0.0248
			Month 6	1.2185	0.1311	0.4659	0.7847
		*[Clostridium] hiranonis*	Baseline	7.8269	6.5843	0.2936	0.8542
			Month 1	6.6705	4.3463	0.1152	0.4095
			Month 3	8.1707	11.122	0.4622	0.6724
			Month 6	12.4726	11.1433	0.6744	0.8286
		*Romboutsia ilealis*	Baseline	11.9025	19.6981	0.1722	0.7218
			Month 1	0.3433	0.8364	0.0503	0.2787
			Month 3	0.1771	1.7572	0.0033	0.0262
			Month 6	1.9804	0.804	1	1
		*Romboutsia UC*	Baseline	6.9703	3.0065	0.1722	0.7218
			Month 1	0.3298	0.1052	0.4659	0.7847
			Month 3	0.161	0.5972	0.0569	0.1708
			Month 6	3.2744	0.1568	1	1
	Ruminococcaceae	*Faecalibacterium prausnitzii*	Baseline	0.6445	1.3055	0.6363	0.8633
			Month 1	1.336	3.0624	0.5286	0.8458
			Month 3	0.7991	1.4399	0.9164	0.9774
			Month 6	0.7217	0.2508	0.3805	0.7162
	Selenomonadaceae	*Megamonas funiformis*	Baseline	0.3385	0.1047	0.1722	0.7218
			Month 1	1.7823	2.9722	0.4008	0.7847
			Month 3	1.9208	1.6765	0.4622	0.6724
			Month 6	1.7089	3.3438	0.6744	0.8286
	Streptococcaceae	*Streptococcus lutetiensis*	Baseline	0.5142	0.1451	0.125	0.7218
			Month 1	5.5431	8.8683	0.752	0.9774
			Month 3	12.8114	16.0298	1	1
			Month 6	7.832	19.6741	0.0274	0.2194
**Fusobacteriota**	Fusobacteriaceae	*Fusobacterium UC*	Baseline	0.7752	0.9419	0.9161	1
			Month 1	2.9933	3.2687	0.9164	0.9774
			Month 3	2.0075	6.5708	0.0157	0.0838
			Month 6	3.0966	5.3385	0.2936	0.6264
		*Fusobacterium UC2*	Baseline	0.6151	1.1728	0.7525	0.8919
			Month 1	1.3253	2.4665	1	1
			Month 3	1.6845	3.4663	0.7527	0.9199
			Month 6	2.0153	0.4598	0.5984	0.8286
**Proteobacteria**	Enterobacteriaceae	*Escherichia coli*	Baseline	0.0479	0.071	0.2016	0.7218
			Month 1	0.0411	0.1611	0.9127	0.9774
			Month 3	0.1509	0.8847	0.0154	0.0838
			Month 6	0.2397	5.0395	0.0355	0.2271
	Sutterellaceae	*Sutterella stercoricanis*	Baseline	0.4402	0.2621	0.9578	1
			Month 1	0.5227	0.7402	0.5995	0.872
			Month 3	0.7078	0.4594	0.4005	0.6724
			Month 6	0.3473	0.2081	0.2476	0.6264

1 Only bacterial genera with mean relative abundances >1% across the entire dataset are shown.

Butyrate was the only SCFA affected by diet (*P* < 0.01), with dogs fed the fresh diet having greater fecal concentrations overall (fresh: 72.94 ± 10.23 µmol/g DM; kibble: 48.74 ± 6.83 µmol/g DM). It should be noted that there was a tendency for the two groups to differ at baseline before feeding either test diet when baseline data was assessed separately (fresh: 63.36 ± 10.33 µmol/g DM; kibble: 49.51 ± 8.08 µmol/g DM; *P* = 0.06). When analyzing the diet*time interaction with Tukey’s post-hoc adjustment in the mixed model, there were no differences between the groups at baseline (*P* = 0.64), but there were differences between the treatments at the end of study (fresh: 92.30 ± 15.05 µmol/g DM; kibble: 49.30 ± 8.04 µmol/g DM; *P* < 0.01). There was a diet*time interaction for valerate (*P* = 0.05), in which dogs on the kibble diet increased by 3 months compared to baseline but then declined to intermediate levels by 6 months. Valerate in the fresh diet did not change during this time. Propionate increased across both diets by 6 months (*P* < 0.01). No differences or changes were observed in other fecal characteristics or BCFA.

### Fecal microbiome

Transitioning from the baseline diet to both the fresh and kibble diets significantly increased alpha diversity (*P* < 0.05). However, no statistically significant differences in alpha diversity were detected between the fresh and kibble diets at any time point (*P* > 0.05). After 1 mo on the study diets, the fecal microbiomes of the fresh and kibble groups diverged, indicating that diet played a role in shaping microbial composition (PERMANOVA Aitchison, R2 = 0.14, *P* = 0.01; Bray–Curtis R2 = 0.07, *P* = 0.36). By the 3-mo timepoint, this divergence became even more pronounced, with clear differences in the composition of the fecal microbiome between the two diets (PERMANOVA Aitchison, R2 = 0.18, *P* = 0.001; Bray–Curtis R2 = 0.17, *P* = 0.007). This difference persisted through mo 6 (Figure 2).

**Figure 2:**
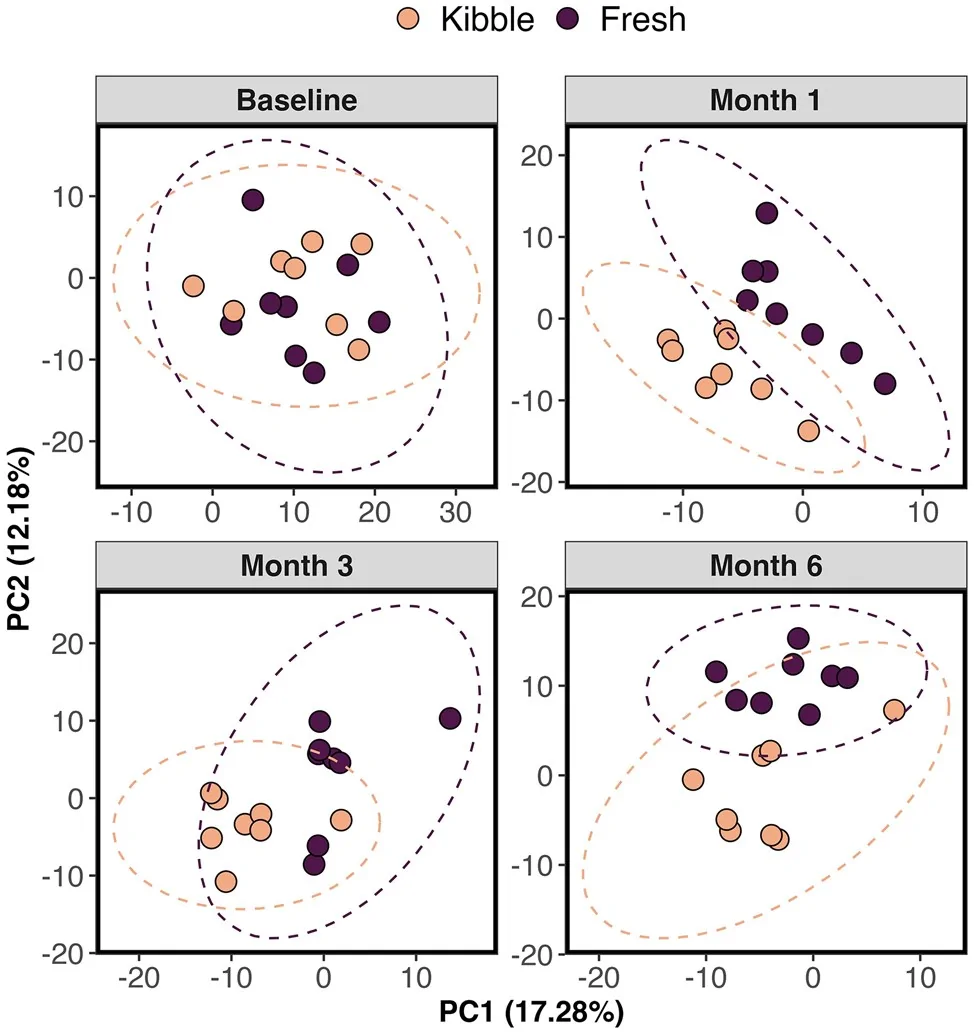
PCoA ordinations derived from Aitchison distances (CLR-transformed abundances) demonstrate separation of microbial communities by diet (kibble vs. fresh) at baseline and 1, 3, and 6 mo post-transition.

Differential abundance analysis was performed to identify which bacterial species were driving differences in microbiome beta diversity between the two diet groups. At 3 months, dogs fed the fresh diet had greater relative abundance of *Romboutsia ilealis*, *Clostridium dakarense*, *T. sanguinis*, and *E. coli*, while the kibble group had greater relative abundance of *Lactobacillus* spp., *Bifidobacterium pullorom*, *Holdemanella biformis*, *Prevotella copri*, and *Catenibacterium mitsuokai* (Figure 3, *P* < 0.05). After 6 months, the fresh diet group had greater relative abundance of *Ruminococcus torques, Clostridium perfringens, Lactobacillus animalis*, and *Escherichia coli* compared to dogs fed kibble. The kibble group had greater relative abundance of *H. biformis, Parabacteroides* UC (unclassified), and *C. mitsuokai* (Figure 4, *P* < 0.05).

**Figure 3:**
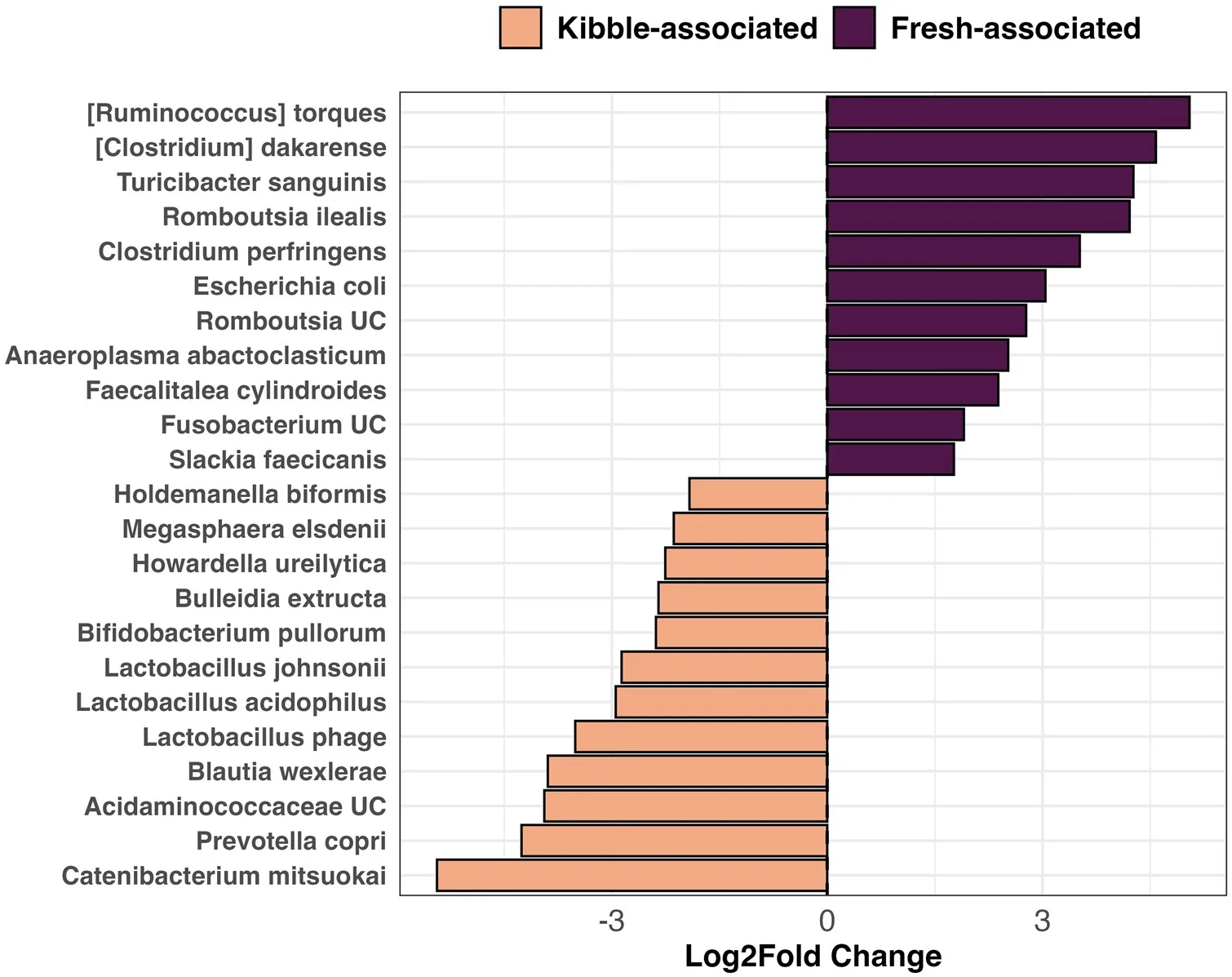
Bacterial species enriched in the fecal microbiomes of dogs an extruded dry kibble diet vs. a fresh diet 3 mo after diet transition. Differential abundance was assessed using LinDA analyses at the species level (*P* < 0.05).

**Figure 4:**
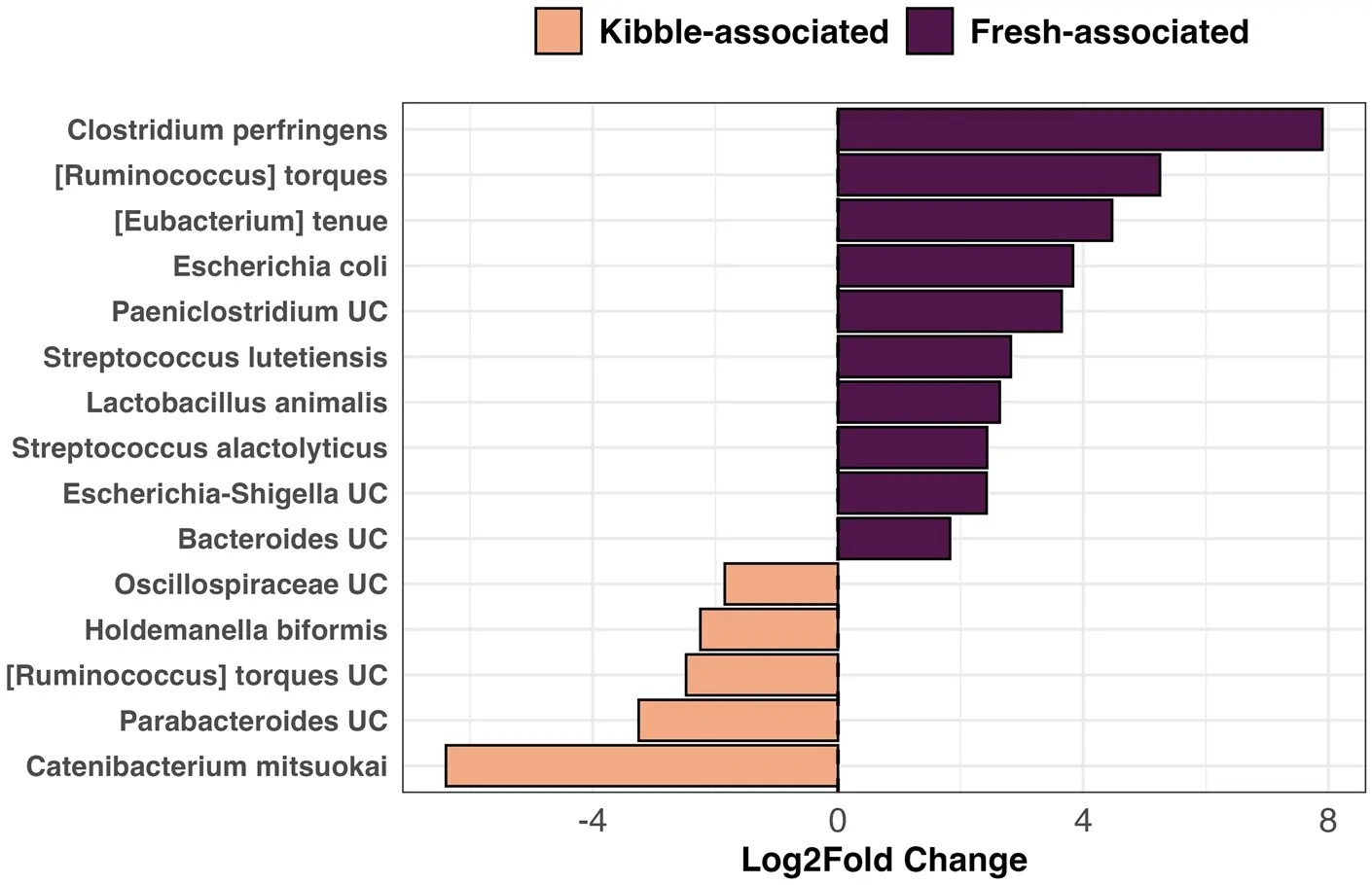
Bacterial species enriched in the fecal microbiomes of dogs fed an extruded dry kibble diet vs. a fresh diet 6 mo after diet transition. Differential abundance was assessed using LinDA analyses at the species level (*P* < 0.05).

In addition to differential abundance analysis, Wilcoxon tests (*P* < 0.05) compared the relative abundances of 29 bacterial species (Figures 5 and 6) between the two diet groups at each time point. At mo 1, fresh diet dogs appeared to harbor more *Clostridium disporicum*, *C. perfringens*, and *R. ilealis*, while kibble dogs had more *C. mitsuokai* and *Peptococcus* UC, though the *P* values become non-significant (*P* < 0.05) after FDR correction.

**Figure 5:**
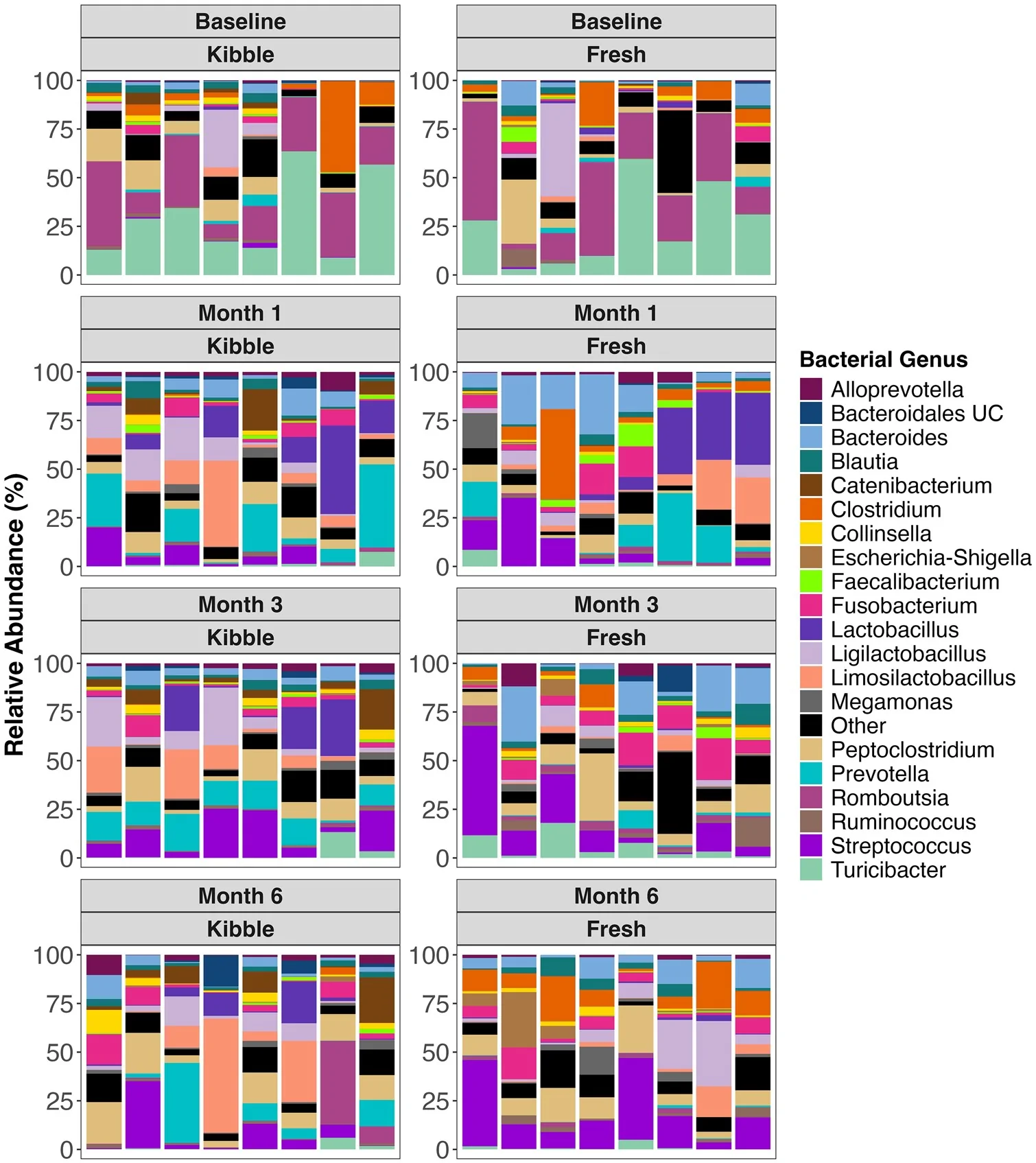
Relative abundances of bacterial genera in the fecal microbiomes of dogs fed an extruded dry kibble diet or a fresh diet at four study timepoints. Bacterial genera with mean relative abundances >1% across all samples are displayed, while all other taxa are grouped under the category “Other.”

**Figure 6:**
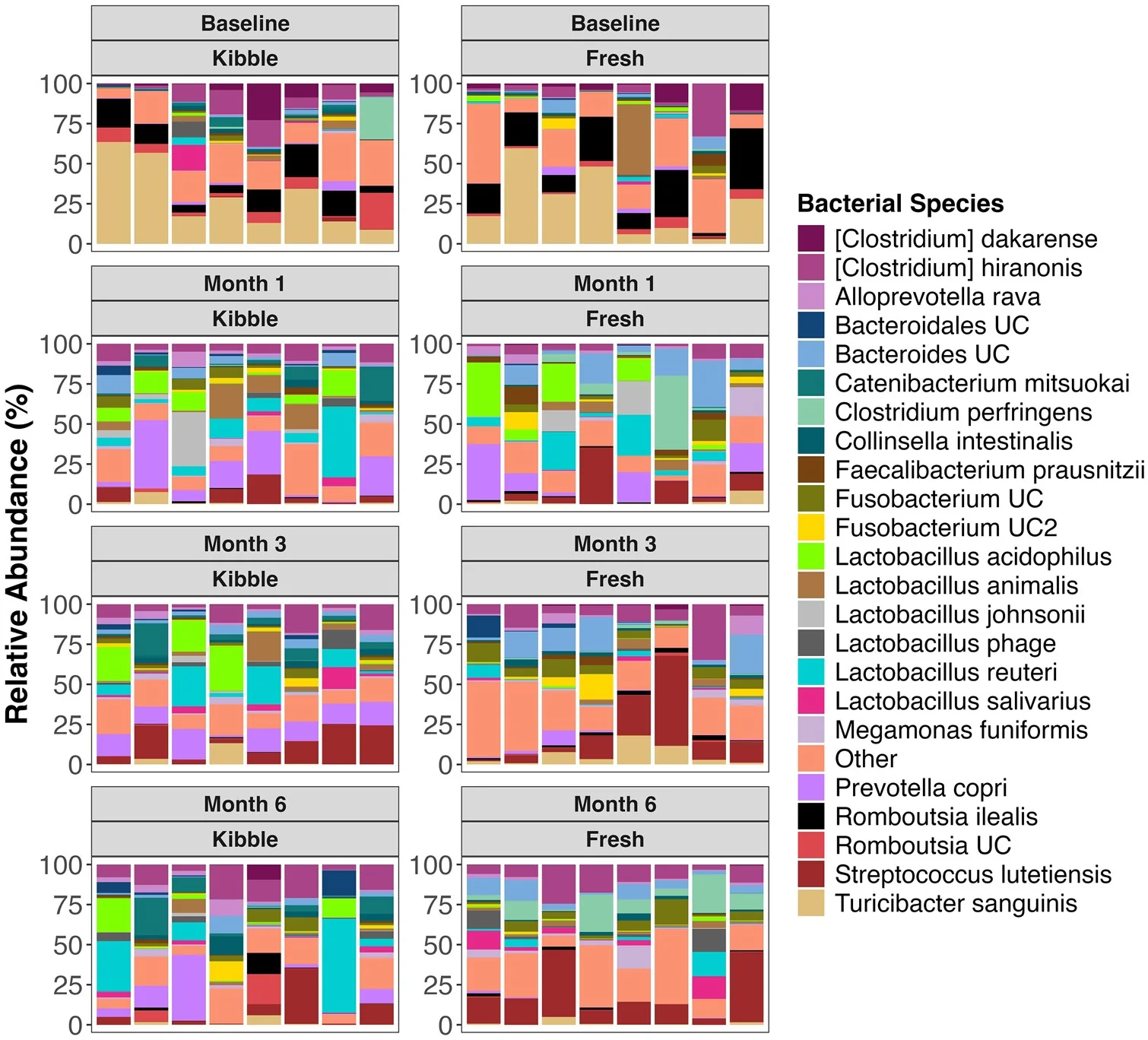
Relative abundances of bacterial species in the fecal microbiomes of dogs fed an extruded dry kibble diet or a fresh diet at four study timepoints. Bacterial species with mean relative abundances >0.9% across all samples are displayed, while all other taxa are grouped under the category “Other.”

At mo 3, dogs consuming the fresh diet had more *C. dakarense, E. coli, Fusobacterium* UC, and *T. sanguinis*, while dogs consuming the kibble diet had more *Lactobacillus acidophilus, P. copri*, and *C. mitsuokai*. Patterns involving *C. dakarense*, *P. copri*, and *C. mitsuokai* remained significant (*P* < 0.05) after FDR correction.

By mo 6, many of the differences detected in mo 3 were no longer observed. The fecal microbiomes of dogs fed the fresh diet continued to have more *Escherichia* and *C. perfringens*, as well as increased *Streptococcus*, but these patterns were not statistically significant for *E. coli* and *Streptococcus* after FDR correction (*P* > 0.05). The relative abundances of these groups increased from mo 3 to mo 6 (Table 3).

## Discussion

As fresh dog diets become increasingly popular, a better understanding of their impact on canine health is needed. The aim of this study was to evaluate and compare the effects of a fresh-frozen diet and a kibble diet on the fecal health and microbiome of dogs after long-term feeding. While both diets were chicken-based and were able to support canine health over the 6-mo trial, they differed in macronutrient content and ingredients, which likely contributed to the differences observed between the two diets, particularly in the microbiota.

Dogs consuming the fresh diet had improved fecal scores compared to those fed kibble. The fresh group had firmer (but not too hard) stools. This divergence was observed quickly, as significant differences were detected within the first 10 d after beginning the transition to the study diets. Previous studies observed no difference in fecal quality between fresh and kibble groups; however, they only compared diets at one timepoint and no baseline fecal scores were reported (Algya et al. 2018; Do et al. 2021), so it is not clear if the groups had different fecal quality before consuming the test diets. These studies also only used Beagles. There are differences in the structure and physiology of the gastrointestinal tracts of small versus large dogs (Deschamps et al. 2022). Large breed dogs like Labrador Retrievers have a longer large intestine transit time, meaning ingested matter spends more time in the large intestine (Hernot et al. 2006; Weber et al. 2017). A longer transit time allows for more time for the fermentation of residual protein and fiber to occur (Korpela 2018; Procházková et al. 2023). Differences in gut structure and function between large and small breeds may have contributed to the observed differences in fecal consistency among our dogs compared to those of previous studies.

The type and amount of fiber included in a diet can also influence fecal consistency and microbiome. Of the first five ingredients in the kibble diet, two are protein sources and three are cereal grains (brown rice, oatmeal, and barley). In the fresh-frozen diet, one of the first five ingredients is a cereal grain (rice) and two others are vegetables (carrots and peas). Cereal grains are higher in insoluble, nonfermentable fibers such as cellulose and lignin, while fruits and vegetables are higher in soluble, fermentable fibers like pectin. Barley and oats are also high in beta-glucan, a soluble fiber (McBurney and Thompson 1990; Mysonhimer and Holscher 2022; Montserrat-Malagarriga et al. 2024). Soluble fibers, like those of fruits, vegetables, and barley and oats, increase small intestine transit time. Insoluble fibers, like those of cereal grains, decrease transit time in the small and large intestines (Moreno et al. 2022). The kibble diet has a greater ratio of insoluble: soluble fiber than the fresh diet (kibble: 1.77:1; fresh: 1.60:1), which may help explain the differences in fecal quality. There is also substantially greater crude and total dietary fiber in the kibble diet compared to the fresh diet, which may contribute to less colonic fermentation in the large intestine. Observing transit time alongside fecal quality in future studies would help elucidate the role fiber type plays in stool consistency.

Along with fiber, protein also plays a role in fecal consistency and the gut environment. German Shepherds fed protein sources of differing digestibility and varied protein levels had reduced fecal consistency with high levels of lesser digestible protein, as well as increased protein-based fermentation products (Nery et al. 2010). While digestibility and fecal protein content were not measured in this study, the lack of differences in the other fecal parameters may be indicative of differences between the fresh and kibble diets’ protein digestibility. The fresh group’s firmer stool may be a sign of less protein being present in their stool (Jackson et al. 2024a). In addition, the tested protein-based fermentation products ammonia and BCFA were not significantly different between the two diet groups. Again, while digestibility of the diets was not a primary focus in this study, this association between fecal protein, ammonia, BCFA, and stool quality contributes to previous studies’ evidence for the increased digestibility of protein in fresh dog food (Algya et al. 2018; Geary et al. 2024; Jackson et al. 2024a). The lack of digestibility information is a limitation of this article. Future studies should measure diet digestibility, as well as stool volume, as this can be impacted by fiber content and nutrient digestibility.

A major limitation of this article was the difference in nutrient content between the kibble and fresh diets. The fresh diet was greater in protein, fat, and ash and was lower in fiber. The largest percent difference of nutrients between diets was that of fiber and protein. As these both affect stool quality and the microbiome, the observed changes in this article may be due to protein and fiber content rather than diet preparation method (Ephraim et al. 2020; Do et al. 2021; Phungviwatnikul et al. 2021; Geary et al. 2024). Future studies should consider formulating extruded diets to match the nutrient content of their fresh diets, especially as the current literature is lacking studies comparing novel diet formats that have matched macronutrient levels. While comparing diets matched in nutritional content would address the role that diet preparation method alone plays in affecting gut health, the diets in this article were chosen to reflect the real-world decisions consumers face when purchasing dog food and deciding between premium kibble or fresh food. However, the authors acknowledge that more prudent discerning of the nutrient profiles would have been beneficial for this article. In addition, inherent constraints associated with the extrusion process may limit the ability to precisely match the ingredient and nutrient composition of a fresh-frozen diet.

The fecal microbiomes of dogs fed the fresh diet were significantly different from those fed the kibble diet, as determined via beta diversity analysis; previous studies observed similar results. Algya et al. (2018) fed dogs either a frozen raw, freeze-dried raw, fresh, or extruded diet, and observed that each group had a distinct microbiome that differed from all other groups. Geary et al. (2024) observed a tendency for dogs fed kibble to have a microbiome that differed from dogs fed various fresh diets. However, while these studies observed reduced alpha diversity in dogs fed fresh diets, there was no difference in the alpha diversity of the present study’s fresh and kibble diet groups.

A limitation of this article is the relatively small sample size, which limited statistical power for the Wilcoxon tests comparing the relative abundances of the 32 taxa between the extruded kibble and fresh diet groups. Although several taxa were nominally significant, few remained significant after FDR correction, indicating that the study may have been underpowered to detect differences after accounting for multiple comparisons. Therefore, our ability to interpret species-level differences is limited. We note that fecal samples were processed and sequenced in three batches for the microbiome analysis, which limits this study as it could introduce unintended variability between batches. However, previous validation of this sequencing workflow demonstrated negligible technical variation attributable to sequencing batch (Rojas et al. 2026).

The fresh group had greater relative abundance of *C. dakarense, E. coli*, and *T. sanguinis*, among others, at the 3-mo mark, while the kibble group had greater relative abundance of *L. acidophilus, P. copri*, and *C. mitsuokai*. After 6 months, however, the only significant differences were greater relative abundance of *E. coli, C. perfringens*, and *Streptococcus* in the fresh group. The reasons for changes in certain species from the 3- to 6-mo mark are unclear. It should be noted that samples at each timepoint were processed in batches, which introduces variability and may explain some of the differences. While not suspected based on observations of animal behavior, coprophagia would also play a role, however we would expect more sudden changes between months 1 and 3 as well. Seasonality may also have come into play; studying the gut microbiome of laboratory mice, Matsumoto et al. (2023) observed day length and temperature impacting the make-up of the gut microbiome (Matsumoto et al. 2023). Keeping diet consistent, they artificially altered the mice’s environment to simulate summer or winter conditions and observed differences in the relative abundance of various bacterial taxa between the two groups. Research into the canine fecal microbiome rarely takes place over an extended period of time; natural long-term variation in the dog gut microbiome, to the authors’ knowledge, has yet to be thoroughly investigated, particularly on a monthly or seasonal basis. The gut and fecal microbiomes are complex ecosystems that are influenced by environmental factors including, but not limited to, temperature, humidity, sunlight, the effects of season on behavior, and seasonal infections (Cocomazzi et al. 2025). Season also affects the soil microbiome; temperature and precipitation affect which microbes are able to thrive in the soil (Jansson and Hofmockel 2020; Cornell et al. 2022). The dogs are outside nearly every day, so contact with the changing soil microbiome may have impacted their gut microbiome (Ottman et al. 2019). Recent research observed substantial seasonal changes in pet dogs’ microbiomes as observed via monthly sampling across 1–2 yr (Rojas et al. 2026). All bacterial species investigated showed seasonal variability. Some seasonal fluctuations observed differ from the current study; *Fusobacterium* abundance increased in mo 3 (March) in the dogs fed the fresh diet, while Rojas et al. observed increases in *Fusobacterium* in late summer. As the two groups in this article experienced different shifts over time, perhaps a season*diet interaction factors into the composition of the dog gut microbiome. The marginal fluctuation in body weight during the second half of the study may have influenced the microbiome as well. Obese beagles housed in the same environment and maintained on the same food as those with ideal body condition scores have distinct differences within their microbiomes (Kim et al. 2023). The transition from winter to summer likely caused a slight change in nutrient requirements, however, body weights did not differ from first to last week of the study (*P *= 0.87) by post-hoc analysis and any change was not substantial enough to push dogs out of the ideal body condition range.

In the fermentation of complex carbohydrates and fiber, gut microbes produce SCFA (acetate, propionate, butyrate). SCFA function in myriad ways in the gut, including but not limited to: inhibiting pathogen growth, protecting and maintaining the integrity of the intestinal epithelium, and regulating gut motility (Deng and Swanson 2015; Kim et al. 2025; Yang et al. 2025). Primary SCFA producing gut bacterial phyla include *Firmicutes*, *Bacteroidetes*, and *Actinobacteria*. Previous studies observed no differences in the relative abundance of *Firmicutes* in the fecal microbiomes of dogs fed fresh food or extruded kibble (Algya et al. 2018; Do et al. 2021; Geary et al. 2024). Algya et al. (2018) observed a lower relative abundance of Actinobacteria in dogs fed a fresh diet, and Geary et al. (2024) reported fresh diets raising *Bacteroidetes* relative abundance (Algya et al. 2018; Geary et al. 2024).

At the species level, *P. copri* is an important SCFA producer and one of the most prevalent microbes in the canine fecal microbiome (Rojas et al. 2024). While no SCFA levels were greater in the kibble group, *P. copri* was significantly greater in the kibble group. At the genus level, previous studies have also observed increased relative abundance of *Prevotella* in dogs fed kibble compared to those fed fresh food (Do et al. 2021; Geary et al. 2024). *P. copri* has been observed to be strongly associated with diet, being greater in people with plant-based diets (Ley 2016). Dogs fed a high fiber diet also had greater levels of *P. copri* than dogs fed a low fiber diet which authors attributed to increased insoluble fiber (Bhosle et al. 2025). Similarly, the kibble diet in this study had greater insoluble fiber than the fresh diet. However, the structure, fermentability, and mechanisms of interaction within the microbiome of these ingredients must be considered as well (Opperman et al. 2025). Fiber source appears to play an important role in the composition of the microbiome. While kibble diets often have less protein and more fiber than fresh diets, these differences alone without taking into consideration fiber source do not explain the changes and differences observed in the dogs’ fecal microbiomes.

In addition to carbohydrates and fiber, microbes can also produce SCFA from protein. The SCFA butyrate in particular is the preferred energy source for the cells of the colon and plays a role in preventing diseases of the gut by several mechanisms. As an energy source of colonocytes, adequate butyrate levels reduce the severity of ulcerative colitis. This SCFA regulates transporters in the intestinal wall, such as that of cholesterol. It prevents colonic cancer by promoting apoptosis in pre-cancerous cells while also promoting the growth of healthy intestinal cells. Butyrate also strengthens the intestinal barrier and regulates motility and transit time (Pryde et al. 2002; Guilloteau et al. 2010; Xiong et al. 2022). The primary proteolytic bacterial genera found in the human large intestine include *Bacteroides*, *Propionibacterium*, *Streptococcus*, *Fusobacterium*, *Clostridium*, and *Lactobacillus* (Ma et al. 2017). The dogs fed the fresh diet had greater abundance of *C. perfringens* at mo 3 and *Fusobacterium* at months 1, 3, and 6. Both of these microbes produce butyrate from protein, of which there is more in the fresh diet (Pilla et al. 2021). *Fusobacterium* abundance may also be impacted by fiber sources; Beagles supplemented with cassava fiber or beet pulp had elevated *Fusobacterium* compared to dogs not supplemented with fiber. Cassava fiber is primarily insoluble, while beet pulp is primarily soluble, yet both led to increased *Fusobacterium* (Souza et al. 2021). The roles that protein, fiber, and the interactions between the two play in influencing the gut and fecal microbiomes are complex, and all should be considered when investigating diets that differ in protein and fiber amounts and sources.

The fresh diet dogs, who had greater relative abundance of butyrogenic bacteria, experienced a numerical increase in their fecal butyrate levels, while that of the kibble dogs did not change; the increased protein in the fresh diet may have driven the proliferation of these bacteria and butyrate in the fresh group. Geary et al. (2024) had similar findings on SCFA, with greater butyrate in the fresh group, however they did not see any differences in *Fusobacterium* or *Clostridium*. While diet can drive differences in SCFA-producing bacteria resulting in changes in SCFA production, this is difficult to determine solely from fecal SCFA concentrations. The amount of butyrate present in the feces can be affected by other factors, such as shifts in butyrate absorption in the gut (Macfarlane and Macfarlane 2012). Butyrate production could remain consistent, but if less is absorbed, more will be present in the feces. In addition, the dogs assigned to the fresh diet tended to have greater fecal butyrate concentrations at baseline before diet transition. The fresh group may have had greater levels of butyrate-producing microbes prior to study start and continued to have greater levels than the kibble group over the six months of this article.

Dogs on the fresh diet had greater relative abundance of *E. coli* at 6 months. This aligns with previous studies that also observed elevated *E. coli* in dogs fed fresh diets compared to those fed kibble (Geary et al. 2024; Oba et al. 2025). Previous studies have also reported an enrichment of *E. coli* in dogs fed raw food compared to dogs fed kibble (Rojas et al. 2024). To the general public, *E. coli* is known as a causative agent of gastrointestinal distress. However, *E. coli* is a common microbe identified in the guts of healthy humans and dogs (Martinson and Walk 2020; Rojas et al. 2024). There are many strains of *E. coli*, and only certain strains cause illness. Non-pathogenic *E. coli* serve several functions in the gut, such as vitamin K production and creating a suitable environment for other beneficial microbes, so simply identifying the presence of *E. coli* in the gut is not concerning (Martinson and Walk 2020). As none of the dogs in this article showed signs of gastrointestinal distress, it is likely that the observed increase was in primarily non-pathogenic *E. coli*. A similar case appears with *C. perfringens*, several strains of which have been reported to be associated with gastrointestinal distress in dogs. However, no studies have confirmed this association, and *C. perfringens* has been classified as a core bacteria of the canine gut microbiome (Silva and Lobato 2015; Rojas et al. 2024). Shedding of pathogenic strains of these bacteria could pose concern for human and pet health as seen through dogs fed raw meat based diets (Ramos et al. 2022). However, fresh-frozen foods are processed to inactivate pathogens and may pose less risk than raw diets. Unfortunately, strain-level microbiome analyses that would allow us to know the pathogenicity of fecal microbes were not performed in this article.

## Conclusion

In conclusion, a fresh-frozen diet improved dogs’ fecal scores compared to a kibble diet, and the transition to fresh food produced measurable improvements in fecal consistency in as little as 1 wk. Both diets tested in this study were well tolerated, and all dogs remained healthy over the course of the study. All dogs experienced an increase in alpha diversity after transitioning from the kennel diet to either test diet. Fresh-frozen food drove a distinct microbiome profile, fostering microbial communities that may have contributed to the production of butyrate, and may offer additional health benefits, which warrant further study. These differences may be attributed to factors such as nutritional content and processing methods. Future studies should consider using dog foods that are identical in nutrient content to compare diet types and better elucidate differences in canine gut health and fecal quality due solely to processing method.

## Supplementary Material

skag303_Supplementary_Data

## Supplementary data

*Conflict of interest statement.* Makenzie C. Melby, Claire L. Timlin, and Fiona B. McCracken are employees of Four Rivers Kennel, Walker, MO, United States at the time of this study. Craig N. Coon is the President and Co-Founder of Four Rivers Kennel. Connie A. Rojas is an employee at Animal Biome. Amanda Blake is an employee of the Gastrointestinal Laboratory, Texas A&M University which provides diagnostic and research testing on a fee-for-service basis. Catherine Carchedi, Brittany White, and Lindsay Cassibry are employees of Ollie Pet Inc. and played a role in funding, study design, write-up, and final review of the manuscript, and decision to publish. The authors declare no other conflicts of interest.

## Abbreviations

BCS: body condition score
DM: dry matter
SCFA: short-chain fatty acid
