# Supplementary material for: Impacts of a fresh-frozen dog food on fecal consistency and microbiome in colony dogs
Source: J Anim Sci. 2026 Sep 21;104:skag303. doi: 10.1093/jas/skag303 (PMC13645448; doi:10.1093/jas/skag303)
Supplement: Supplementary file 1 [file skag303_supplementary_data.docx]

**Supplementary Materials**

**Supplementary Materials S1.** Basal kennel diet, MFA Gold N’ Pro, information.

Ingredients: Poultry byproduct meal, ground yellow corn, corn distiller’s dried grains with solubles, poultry fat (preserved with BHA and BHT)< pearled barley, pork meal, dried plain beet pulp, natural poultry flavors, whole flaxseed, salt, potassium chloride, choline chloride, ferrous sulfate, zinc sulfate, copper sulfate, manganese sulfate, vitamin E supplement, niacin supplement, calcium pantothenate, biotin, vitamin A supplement, thiamine mononitrate, riboflavin, pyridoxine hydrochloride, vitamin B12 supplement, vitamin D3 supplement, folic acid, sodium selenite, calcium iodate, and cobalt carbonate

Guaranteed Analysis:

Crude protein (min): 27%

Crude fat (min): 15%

Crude fiber (max): 3%

Moisture (max): 10%

Omega 6 fatty acids (min): 2.4%

Omega 3 fatty acids (min): 0.3%

Calorie Content: ME (calculated): 3515 kcal/kg, 395 kcal/cup

**Supplementary Table 1.** Average body weights and body condition scores ± SEM. Ideal body condition score range: 4 to 5.

|  | Body Weight (kg) | | Body Condition Score | |
| --- | --- | --- | --- | --- |
| Week | Kibble | Fresh | Kibble | Fresh |
| 0 | 25.90 ± 1.34 | 26.35 ± 1.29 | 4.06 ± 0.16 | 4.37 ± 0.16 |
| 1 | 26.04 ± 1.32 | 26.25 ± 1.30 | 4.12 ± 0.16 | 4.37 ± 0.16 |
| 2 | 26.03 ± 1.32 | 26.49 ± 1.28 | 4.12 ± 0.16 | 4.31 ± 0.16 |
| 3 | 26.08 ± 1.32 | 26.76 ± 1.25 | 4.18 ± 0.16 | 4.56 ± 0.16 |
| 4 | 26.24 ± 1.30 | 26.87 ± 1.24 | 4.25 ± 0.16 | 4.62 ± 0.16 |
| 5 | 26.25 ± 1.30 | 26.74 ± 1.25 | 4.12 ± 0.16 | 4.5 ± 0.16 |
| 6 | 25.96 ± 1.33 | 26.78 ± 1.25 | 4.12 ± 0.16 | 4.5 ± 0.16 |
| 7 | 26.18 ± 1.31 | 27.03 ± 1.23 | 4.25 ± 0.16 | 4.56 ± 0.16 |
| 8 | 25.98 ± 1.33 | 26.73 ± 1.26 | 4.25 ± 0.16 | 4.75 ± 0.16 |
| 9 | 26.29 ± 1.30 | 26.99 ± 1.23 | 4.37 ± 0.16 | 4.56 ± 0.16 |
| 10 | 26.13 ± 1.31 | 26.90 ± 1.24 | 4.18 ± 0.16 | 4.56 ± 0.16 |
| 11 | 26.16 ± 1.31 | 26.40 ± 1.29 | 4.31 ± 0.16 | 4.5 ± 0.16 |
| 12 | 26.60 ± 1.27 | 26.42 ± 1.29 | 4.25 ± 0.16 | 4.56 ± 0.16 |
| 13 | 26.24 ± 1.30 | 25.94 ± 1.33 | 4.12 ± 0.16 | 4.56 ± 0.16 |
| 14 | 26.29 ± 1.30 | 26.36 ± 1.29 | 4.25 ± 0.16 | 4.62 ± 0.16 |
| 15 | 26.27 ± 1.30 | 26.26 ± 1.30 | 4.56 ± 0.16 | 4.81 ± 0.16 |
| 16 | 26.78 ± 1.25 | 26.64 ± 1.26 | 4.62 ± 0.16 | 4.87 ± 0.16 |
| 17 | 27.09 ± 1.22 | 27.00 ± 1.23 | 4.43 ± 0.16 | 4.75 ± 0.16 |
| 18 | 27.26 ± 1.21 | 26.81 ± 1.25 | 4.37 ± 0.16 | 4.68 ± 0.16 |
| 19 | 27.76 ± 1.16 | 27.20 ± 1.21 | 4.37 ± 0.16 | 4.75 ± 0.16 |
| 20 | 27.40 ± 1.19 | 26.95 ± 1.24 | 4.31 ± 0.16 | 4.68 ± 0.16 |
| 21 | 27.74 ± 1.17 | 27.31 ± 1.20 | 4.18 ± 0.16 | 4.75 ± 0.16 |
| 22 | 27.43 ± 1.19 | 26.92 ± 1.24 | 4.18 ± 0.16 | 4.68 ± 0.16 |
| 23 | 28.07 ± 1.14 | 27.58 ± 1.18 | 4.37 ± 0.16 | 4.68 ± 0.16 |
| 24 | 28.01 ± 1.14 | 27.57 ± 1.18 | 4.37 ± 0.16 | 4.68 ± 0.16 |
| 25 | 27.95 ± 1.15 | 27.62 ± 1.18 | 4.43 ± 0.16 | 4.68 ± 0.16 |
| 26 | 27.97 ± 1.15 | 27.06 ± 1.22 | 4.43 ± 0.16 | 4.62 ± 0.16 |

**Supplementary Figure 1.** Body weights in kilograms by week and diet over the course of the study.

Body Weight, kg

| Week | Fresh | Kibble |
| --- | --- | --- |
| 0 | 26.3541 | 25.9005 |
| 1 | 26.2534 | 26.0468 |
| 2 | 26.4959 | 26.0364 |
| 3 | 26.7624 | 26.0801 |
| 4 | 26.8797 | 26.2457 |
| 5 | 26.7471 | 26.2554 |
| 6 | 26.781 | 25.964 |
| 7 | 27.0301 | 26.1822 |
| 8 | 26.7312 | 25.9872 |
| 9 | 26.9909 | 26.2954 |
| 10 | 26.9087 | 26.1383 |
| 11 | 26.4001 | 26.1607 |
| 12 | 26.4216 | 26.6028 |
| 13 | 25.9442 | 26.2413 |
| 14 | 26.3652 | 26.2901 |
| 15 | 26.267 | 26.2752 |
| 16 | 26.647 | 26.7834 |
| 17 | 27.0027 | 27.0934 |
| 18 | 26.814 | 27.2671 |
| 19 | 27.2042 | 27.765 |
| 20 | 26.951 | 27.4045 |
| 21 | 27.3141 | 27.7477 |
| 22 | 26.9289 | 27.4391 |
| 23 | 27.5879 | 28.0704 |
| 24 | 27.5757 | 28.0144 |
| 25 | 27.626 | 27.9596 |
| 26 | 27.0693 | 27.9757 |

**Supplementary Figure 2.** Body condition score by week and diet over the course of the study. Ideal body condition score range: 4 to 5.

Body Condition Score

| Week | Fresh | Kibble |
| --- | --- | --- |
| 0 | 4.375 | 4.0625 |
| 1 | 4.375 | 4.125 |
| 2 | 4.3125 | 4.125 |
| 3 | 4.5625 | 4.1875 |
| 4 | 4.625 | 4.25 |
| 5 | 4.5 | 4.125 |
| 6 | 4.5 | 4.125 |
| 7 | 4.5625 | 4.25 |
| 8 | 4.75 | 4.25 |
| 9 | 4.5625 | 4.375 |
| 10 | 4.5625 | 4.1875 |
| 11 | 4.5 | 4.3125 |
| 12 | 4.5625 | 4.25 |
| 13 | 4.5625 | 4.125 |
| 14 | 4.625 | 4.25 |
| 15 | 4.8125 | 4.5625 |
| 16 | 4.875 | 4.625 |
| 17 | 4.75 | 4.4375 |
| 18 | 4.6875 | 4.375 |
| 19 | 4.75 | 4.375 |
| 20 | 4.6875 | 4.3125 |
| 21 | 4.75 | 4.1875 |
| 22 | 4.6875 | 4.1875 |
| 23 | 4.6875 | 4.375 |
| 24 | 4.6875 | 4.375 |
| 25 | 4.6875 | 4.4375 |
| 26 | 4.625 | 4.4375 |
